# Therapeutic Pearls at Random Strung

**Published:** 1881-03

**Authors:** 


### THERAPEUTIC PEARLS AT RANDOM STRUNG.

### DIARRHŒA AND DYSENTERY.

*Five* grains of *salicine* every four, or six hours, is said to act almost specifically in the above diseases where the tongue is clean, and the symptoms have not yielded to opium, astringents, &c.

### DYSENTERY.

| | | |
|---|---|---|
| ℞ | Carbolic Acid, . . . . . . | grs. 15 |
| | Spt. Wine, Rectified, . . . | M. 15 |
| | Tinct. Opium, . . . . | M. 15 to 20 |
| | Mucilage and Poppy Syrup, . . . | aa ʒ vi |
| | Distilled Water, . . . . . . | ℥ iii M |

A tablespoonful to be taken every two hours. To children, smaller doses, to be given according to age. As much as 7 or 8 grains of Carbolic Acid were given by Dr. Amelung in 24 hours during an epidemic of dysentery.

### IPECACUANHA IN DYSENTERY.

Ipecacuanha is said by Dr. John Stephen, (Medical and Surgical Reporter, Vol. 23, Page 421,) to act in doses of from 30 to 80 grains, *Specifically*, in dysentery. Castor Oil alone, or in combination, may be given and after its action a dose of Ipecacuanha administered. Or opium may be prescribed in a free dose, and an hour or two later the Ipecac administered.

The case may be concluded with the following combination:

| | | |
|---|---|---|
| ℞ | Camphor Water, . . . . . | ℥ ij |
| | Paregoric " . . . . . . | ʒ ij |
| | Comp. Spts, Lavender, . . . . . | ℥ ij M. |

Dose *one* tablespoonful every three hours.

### DIPHTHERIA.

*Lactic Acid* is reported to be a valuable agent in dissolving the diphtheritic membrane. It should be used freely locally. *Ferri per Chloridi*, may be used as a gargle, and *Chlorate of Potash*, used internally.

### Diphtheria.

*Liquor Potasæ*, well diluted with water, has been strongly recommended in Diphtheria. The dose should be 20 drops in a wine-glassful, or two, of water, every three hours to an adult, and the quantity proportioned for children according to age.

### Diphtheria.

*Pure Lemon Juice*, used in large quantities as a gargle has been found always successful for a period of 18 years by M. Revıllont and his father.

Dr. R. H. Thornton, Newport, Kentucky, says:

"For several years past I have been in the habit of using a topical application advised by Smith, of New York, viz:

℞ Carbolic Acid, . . . . . gr. iv

Sol. of subsulphate of iron, . . ʒ j.

Glycerine, . . . . . . ℥ ss

S. Apply with a camel's hair pencil once or twice a day.

Inwardly have had most faith in chlorate of potash, perchloride of iron and sherry wine, aided occasionally by quinine. More recently have been trying, with prompt and most satisfactory results, the prescriptions recommended by Robert Bell:

The one for painting is—

℞ Glycerite of carbolic acid,
Sulphurous acid,
Strong solution of perchloride of iron, *aa* ʒ ij M.

The other is taken inwardly in two teaspoonful doses and at bihoral intervals, and is as follows:

℞ Sulphurous acid, . . . . . ʒ ijss.

Chlorate of potash, . . . . . . ʒ ij.

Glycerine, . . . . . . . ʒ j.

Water q. s. ad., . . . . . . ℥ iv. M.

The latter is taken one hour and the throat painted the next."

### Dropsy.

*A saturated solution of Chlorate of Potash*, given in *teaspoonful doses* every *four* hours, has been found a valuable remedy in *all* dropsical effusions, by Dr. Samuel Peters, of New York State. As appears in the Transactions of the State Medical Society for the year 1870, published at Albany.

### Dyspepsia.

℞ Rennet Wine, . . . . . . . Oj
Tinct. Nux Vomica,
Nitro-Muriatic Acid, Dilute.
Sub-Nitrate Bismuth, . . . *aa* ℥ ij M.
S. A tablespoonful half hour before meals, in water.

### Erysipelas.

*Tinct. Ferri Mur*, applied externally until the skin refuses to absorb more. *Iodide* of *potash* may be given internally in 10 to 20 grain does in syrup or molasses at the same time, every 3 or 4 hours.

*Sulphite of Soda*, in saturated solution, may be applied on cloths previously dipped in it.

*Nitrate of Potash*, may be applied in a similar manner.

*Cranberries*, in the form of poultice have been found highly valuable in certain cases of erysipelas.

### Eczema.

*Tincture of Iodine*, painted over the eruption will often be found highly valuable.

*Hebras Tincture*, a compound of equal parts of *tar*, *soft soap* and *mythilated spirit*, should be applied twice daily, and after having dried on the skin should be washed off with soap or *petroleum* soap.

*Mucilage of Starch or decoction of bran* will be found useful in allaying itching and absorbing discharges.

ECZEMA.

℞ Tinct. Ferri Chloridi, . . . . . ℥ i

Liq. Potassæ Arsenitis, . . . . ʒ iss

Hydrarg Chlor. cor, . . . . . gr. ij M.

S. xxx drops ter die to an adult, and apply locally the ointment of the Oxide of Zinc, below named.

℞ Nuguent, Oxide Zinc, . . . . . ℥ i
Acid Benzoic, . . . . . . ʒ i M.

Apply locally.

℞ Arsenici Iodidi, . . . . . . . grs. iij
Nuguent Stramoni, . . . . . . ℥ i ft. ung.

Apply locally and give ten or fifteen drops of Donovon's solution three times a day.

℞ Tinct Ferri, Chlor. . . . . . f. ℥ i

Dose 20 drops *before* each meal, as an ectropic and to remove anæmia.

℞ Acid Nitro-Chlor. . . . . . . ℥ i

Dose 6 drops, five minutes *after* each meal as an eliminative, etc.,

℞ Pulv. Cupri Sulph. . . . . . . ʒ j

Aquæ, . . . . . . . . . f. Oj

Apply this to the whole diseased surface once every day or two.

The following is recommended very highly in Eczema, Tenia Capitis and Poison oak poisoning, viz:

℞ Carbolic Acid, cryst.

Sodæ Sulphat, . . . . . . *aa* ℈ i

Sulphas Sublim, . . . . . . ʒ i

Adipis, . . . . . . . . . ℥ i M.

S. Apply twice a day, after washing with soap and water.

CURE OF ECZEMA.

℞ Common Tar, . . . . . ℥ iv. grams. 128 00

Mutton Tallow, . . . . . . ℥ j. " 32 00

Olive Oil, . . . . . . . . ℥ ss. " 16 00

Mix and melt, then add Balsam Fir et

Flour of Sulphur, . . *aa* ℥ j. " 32 00

If the scabs are very hard and dry, a poultice should be applied until they are sufficiently soft to remove. Then apply the ointment three times a day with a soft brush, sufficient to just color the surface of the sores. Every third day grease the surface of the sores for the purpose of removing the tar, then wash carefully with saleratus water, (two teaspoonfuls to the pint) avoiding soap of every kind, then apply the ointment as at first. The second or third application will remove all irritation and itching, and in from ten to fifteen days your patient will have as soft and healthy skin as you may desire.

### Ear.

Foreign bodies may be removed from the *Ear* by doubling horse hair into a loop passing it into the ear and turning it about for a moment or two. The body is usually brought out in the first or second attempt.

### Epilepsy.

℞ Zinci Valerianatis, . . . . . gr. ij
Ext. Cannabis Indicæ, . . . . . gr. ½
Ft. pil, No j. three times a day.

Twenty to thirty grains of Sodium Bromide at night to secure rest.

### Ergot.

Is of great value in partial paralysis of the bladder, as well as in hemorrhage from the mucus surfaces, when combined with *strychnia.*

